# Blue-green neutrophilic inclusion bodies with concurrent liver failure as a predictor of imminent death

**DOI:** 10.1515/almed-2022-0060

**Published:** 2022-07-07

**Authors:** Antonio Sierra Rivera, María García Valdelvira, María A. Elia Martínez, Ángeles Férez Martí, Goitzane Marcaida Benito

**Affiliations:** Servicio de Análisis Clínicos Consorcio Hospital General Universitario de Valencia, Valencia, Spain; Servicio de Análisis Clínicos Hospital Universitario Doctor Peset, Valencia, Spain

**Keywords:** green crystals of death, lactic acidosis, severe liver disease

## Abstract

**Objectives:**

Blue-green, birefractory, poorly-defined, cytoplasmic inclusions in some types of leukocytes are an underdiagnosed finding, which composition and clinical significance is not well understood. Inclusions are only found on peripheral blood smear (PBS).

**Case presentation:**

We report the case of a male with a history of chronic disease (hypertension, obesity, dyslipidemia, and kidney failure) admitted to the emergency room (ER) with shortness of breath, who was later transferred to the intensive care unit (ICU). The patient had a torpid disease course and ultimately died of multiorgan failure, including severe liver failure. When his status exacerbated, these inclusions were observed on PBS in conjunction with liver enzyme abnormalities.

**Conclusions:**

This case confirms that blue-green inclusions on PBS in cases of acute severe liver failure with concurrent lactic acidosis should be immediately reported to the medical team, since it is suggestive of critical status and poor prognosis.

## Introduction

Blue-green inclusion bodies found within the cytoplasm of leukocites is a rare finding associated with a high risk for mortality. These inclusions generally appear in neutrophils, although they are also found, to a lesser extent, in monocytes. So far, nine case reports have been published, reporting around 70 cases that presented these inclusions [1]. These cytoplasmic deposits originate from substances such as biliverdin and lipofuscin, although their origin is not clearly understood. This type of inclusions was formerly called “green crystals of death”, since most patients that presented them died within the following 24–48 h.

Patients with a long-term intensive care unit (ICU) stay are at a higher risk of developing multiorgan failure, which may include acute liver failure that results in the occurrence of these inclusions [2]. In addition, the risk of mortality increases when lactic acidosis occurs concurrently to liver failure, since acidosis is associated with higher mortality [3, 4].

We report a case of blue-green secondary to liver failure managed in our hospital.

## Case presentation

A 66-year-old Caucasian male was admitted to the emergency room (ER) of our hospital with symptoms of dyspnea. Medical history included arterial hypertension, dyslipidemia, obesity, and stage IV renal failure.

Due to low oxygen saturation on arrival, the patient was directly admitted to the intensive care unit (ICU), where oxygen therapy and monitoring were delivered. At that moment, a laboratory analysis was ordered, including hematologic profile, hemostasis and biochemistry, chest X-ray, RT-PCR for SARS-COV-2, and serology for different respiratory viruses.

The RT-PCR test for SARS-COV-2 (GeneXpert, Cepheid^®^) was positive. Laboratory findings included elevated levels of creatinine (7.04 ng/mL) and C-reactive protein (26 mg/dL) in parallel to other abnormalities related to COVID-19 infection, which are described in Table 1. Chest X-ray showed bilateral pneumonia with bilateral peripheral alveolar infiltrates. PCR testing for other respiratory viruses such as, respiratory syncytial virus (RSV), rinovirus and adenovirus (PCR multiplex FilmArray, Biomerieux^®^) was negative.

**Table 1:** Hematological and biochemical parameters on admission, the day before inclusions appeared, and the day inclusions were detected.

HEM/BC	Admission	Day 24 (8 am)	Day 24 (12pm)	Day 25 (8 am)	Units	Reference intervals
Leukocytes	9.9	19	48.7	65.2	10^9^/L	3.8–10.8
Neutrophils	8.8	15.5	39.7	43.7	10^9^/L	1.8–7.5
Lymphocytes	0.6	1.3	4.6	3.3	10^9^/L	1.5–4
Monocytes	0.4	1.3	3.5	5.9	10^9^/L	0.2–0.8
Hemoglobin	12.3	8.9	7.9	11.3	g/dL	13.5–18
Platelets	178	233	356	252	10^9^/L	135–350
PB smear			The presence of neutrophil inclusions is not reflected	Cytoplasmic inclusions are observed in neutrophils. Platelet and erythrocyte anisocytosis		
ALT/GPT	28	23	25	1284	U/L	10–45
AST/GOT	–	20	33	1401	U/L	5–35
GGT	–	303	245	319	U/L	8–55
Total Bb	0.5	0.52	0.51	1.15	mg/dL	0.3–1.2
LDH	1288	650	813	6023	U/L	208–378
AP	54	142	111	131	U/L	30–120
Lactate	1.2	0.5	2.2	10.6	mmol/L	0.5–2
Creatinine	7.04	3.93	3.12	2.59	mg/dL	0.67–1.17
Urea	178	245	169	136	mg/dL	17–43
C React Prot	25.9	12.3	10.9	13	mg/dL	0.00–0.5

HEM, hemogram; BC, biochemistry; PB, peripheral blood; ALT, alanine aminotransferase; GPT, glutamate-pyruvate transaminase; AST, aspartate aminotransferase; GOT, glutamate-oxalacetate transaminase; GGT, gamma glutamyltransferase; Bb, bilirubin; LDH, lactate dehydrogenase; AP, alkaline phosphatase; C Reac Prot, C-reactive protein.

A week after admission, stage IV kidney disease progressed to stage V, and continuous renal replacement therapy (CRRT) was initiated. In the ICU, blood cultures were positive for *Candida auris* and *Staphylococcus aureus*, and treatment with Isavuconazole and Daptomycin was established. Sputum culture grew *Pseudomonas aeruginosa* and *Stenotrophomonas maltophilia*, which were treated with Piperacillin/Tazobactam and Tobramycin. In the third week in the ICU, the patient had a cardiac arrest that was resuscitated. Post-arrest CT scan demonstrated pericardial and pleural effusion. Due to hemodynamic instability, the patient needed 22 transfusions during the two months of hospitalization.

After 25 days in hospital, the daily control hemogram (DxH 800 de Beckman Coulter^®^) demonstrated left shift leukocytosis, monocytosis, myeloid blasts, hemoglobin of 11.3 g/dL and a platelet count of 252 × 10^9^/L. May-Grünwald (MG) staining of peripheral blood smear was performed.

**Figure 1:**
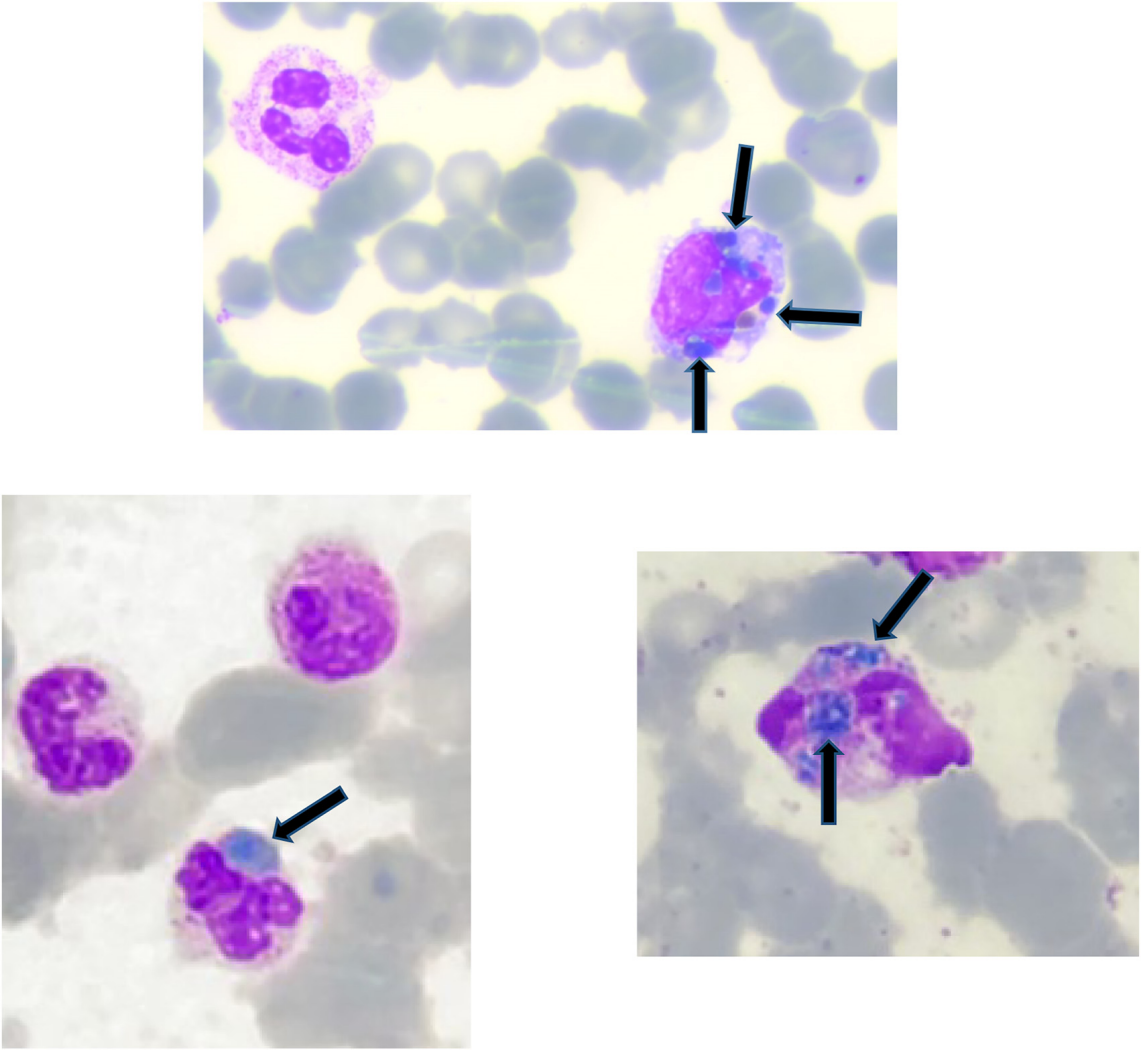
Blue-green cytoplasmic inclusions in peripheral blood smear (May-Gründwald Giemsa).

PBS confirmed left shift leukocytosis (67% segmented, 11% band cells, 1% metamyelocytes, 6% myelocytes). Blue-green, poorly-defined, refractive cytoplasmic inclusions were observed in 1–2% of neutrophils and in some monocytes (Figure 1).

Biochemistry revealed elevated levels of ALT and lactate, and the laboratory decided to extend liver enzyme test to determine aspartate aminotransferase (AST), gamma glutamyltransferase (GGT), bilirubin (Bb), and alkaline phosphatase, apart from lactate dehydrogenase (LDH). Results are shown in Table 1 which contain the course of biochemical and hematological parameters the day of admission, the day before inclusions appeared, and the day they were detected.

Table 1 shows a very significant elevation of ALT/AST and LDH concentrations coinciding with liver failure. Of note, biochemical values remained within normal range or slightly elevated the day before liver failure, and it was not until liver failure occurred, that most parameters increased dramatically.

The laboratory reported the presence of crystals on peripheral blood smear immediately to the responsible physician. Unfortunately, the patient died a few hours later of multiorgan failure.

## Discussion

The precise origin, morphology and composition of these blue-green inclusions are not fully understood [5, 6]. Some hypotheses have been suggested. In acute liver failure, these inclusions may be secondary to blood-borne bile products or residues of lysosomal digestion by neutrophils and monocytes. An example is the green pigment known as biliverdin. In contrast, other authors believe that these crystals originate from a lipofuscin-like product released from necrotic hepatic parenchymal cells [3, 6], [7], [8].

It is important that clinicians are aware of the occurence and clinical significance of these inclusions and are able to differentiate them from other inclusions found within the cytoplasm of neutrophils, such as Döhle bodies, Howell–Jolly pseudobodies, cryoglobulins, and May–Hegglin anomaly, to name a few.

The clinical significance of this finding is uncertain, although it seems to be strongly associated with severe acute liver failure. The small case series available in the literature reveal that patients die shortly after these crystals are observed [7].

Other authors report that 80% of patients with early-stage SARS-COV-2 infection exhibit lymphocytopenia, which returns to normal values after inclusions are detected. As shown in Table 1, the case reported here supports this hypothesis [9].

Peripheral blood smear is crucial and complements hemogram data, since some morphological aspects, such as the presence of these crystals, cannot be detected with hematology analyzers. Microscopic evaluation in the laboratory provides essential information to determine the clinical status of a patient and select potentially useful complementary studies, such as further liver enzyme and LDH testing in this case, to better understand the underlying condition, its course and prognosis.

Further studies are warranted to determine the composition of these crystals. It is also essential that all cases are reported and images are provided for laboratory medicine specialists to learn how to recognize these crystals.

This case supports the evidence provided in the literature and reminds us that the presence of blue-green inclusions on peripheral blood smear of patients with acute liver failure and concurrent lactic acidosis is a predictor of mortality in critically-ill patients with liver insufficiency [4, 10]. The information provided by the laboratory is crucial to establish prognosis.

## Lessons learned

– Blue-green inclusions known as “green crystals of death” are underdiagnosed.
– It is essential that the laboratory immediately reports this finding to alert on the critical status of the patient.
– These inclusions are secondary to acute liver failure associated with lactic acidosis and can only be detected on peripheral blood smear.
